# Development of a robust UPLC-MS/MS method for the quantification of riluzole in human plasma and its application in pharmacokinetics

**DOI:** 10.3389/fphar.2023.1227354

**Published:** 2023-08-09

**Authors:** Zhuo Sun, Xin Liu, Wei Zuo, Qiang Fu, Tingting Xu, Liying Cui, Bo Zhang, Ying Peng

**Affiliations:** ^1^ Department of Pharmacy, Peking Union Medical College Hospital, Chinese Academy of Medical Sciences and Peking Union Medical College, Beijing, China; ^2^ State Key Laboratory of Complex Severe and Rare Diseases, Peking Union Medical College Hospital, Beijing, China; ^3^ State Key Laboratory of Bioactive Substances and Functions of Natural Medicines, Institute of Materia Medica, Chinese Academy of Medical Sciences and Peking Union Medical College, Beijing, China; ^4^ Department of Neurology, Peking Union Medical College Hospital, Chinese Academy of Medical Sciences and Peking Union Medical College, Beijing, China; ^5^ Neuroscience Center, Chinese Academy of Medical Sciences and Peking Union Medical College, Beijing, China

**Keywords:** riluzole, clobazam, amyotrophic lateral sclerosis, ultraperformance liquid chromatography-tandem mass spectrometry, pharmacokinetics

## Abstract

**Introduction:** The aim of the present study was to establish a simple method for the determination of riluzole in human plasma by ultraperformance liquid chromatography-tandem mass spectrometry (UPLC-MS/MS) and apply it for the determination of riluzole in amyotrophic lateral sclerosis (ALS) patients.

**Methods:** Samples were prepared by protein precipitation and were then gradient-eluted on a column of ACQUITY UPLC^®^ HSS T3 by using 0.1% formic acid acetonitrile and 0.1% formic acid water as the mobile phase. Detection was performed on a Xevo TQ-S tandem mass spectrometer in the multiple-reaction monitoring mode using positive electrospray ionization. Validation was performed in the range of 5–800 ng/mL.

**Results and discussion:** Three batches of precision accuracy, selectivity, matrix effects, extraction recovery, and stability were also verified and met the requirements. The results showed that the method was reliable and successfully applied to the pharmacokinetics study of riluzole in Chinese amyotrophic lateral sclerosis patients. Meanwhile, in comparison with other prior published methods, our method has the advantages of simple sample preparation, relatively short running time, and small plasma sample consumption, which represented a high-throughput sample determination potential.

## 1 Introduction

Riluzole is an anti-glutamate drug that has been used to treat amyotrophic lateral sclerosis (ALS) since it was approved by the United States Food and Drug Administration in 1996 (RILUTEK^®^, tablets) (Fehlings et al., 2021). ALS is a rare disease in which patients typically have a survival of only 3–5 years, due to respiratory failure (Grad et al., 2017). In addition, riluzole is the the only oral drug for ALS treatment in China that can prolong the survival of patients by approximately 3 months (Miller et al., 2012). However, the pharmacokinetic characteristics of riluzole in Chinese ALS patients are unknown. Therefore, the goal is to establish a simple method for determining riluzole in humans, which is convenient for the subsequent construction of population pharmacokinetics (popPK) models of Chinese ALS patients to describe their pharmacokinetics characteristics (Bruno et al., 1997).

So far, several methods have been reported for quantitative determination of riluzole in plasma (Sarkar et al., 2017; Chandu et al., 2010; Chow et al., 2012; Le Liboux et al., 1997; van Kan et al., 2004). These methods used either high-performance liquid chromatography (HPLC) with UV detection or ultraperformance liquid chromatography-tandem mass spectrometry (UPLC-MS/MS). The lower limits of quantification (LLOQ) of the HPLC-UV methods described by Le Liboux, van Kan HJ, and Chow DS were 10 ng/mL, 20 ng/mL, and 20 ng/mL, respectively, and all required large amounts of human plasma (1 mL, 500 μL, and 200 μL) (Chow et al., 2012; Le Liboux et al., 1997; van Kan et al., 2004). More recently, Mahua Sarkar and Chandu BR developed the UPLC-MS/MS method for the riluzole assay in human plasma with an LLOQ of 0.5 ng/mL, but all required 200 μL human plasma (Chandu et al., 2010; Sarkar et al., 2017). In addition, the pre-treatment processes for these methods were all liquid–liquid extraction or solid-phase extraction (SPE), which were considered complex.

In this paper, a novel UPLC-MS/MS method was developed. The plasma sample was prepared using protein precipitation, which was simpler than liquid–liquid extraction or SPE, the analytes were highly efficient in a relatively short time on the T3 column, and the volume of the plasma sample used was small. The combination of the three features resulted in a robust and high-throughput analysis method. In addition, the validated analytical method was successfully applied to the quantitative determination of riluzole in Chinese ALS patients.

## 2 Materials and method

### 2.1 Chemicals and regents

**FIGURE 1:**
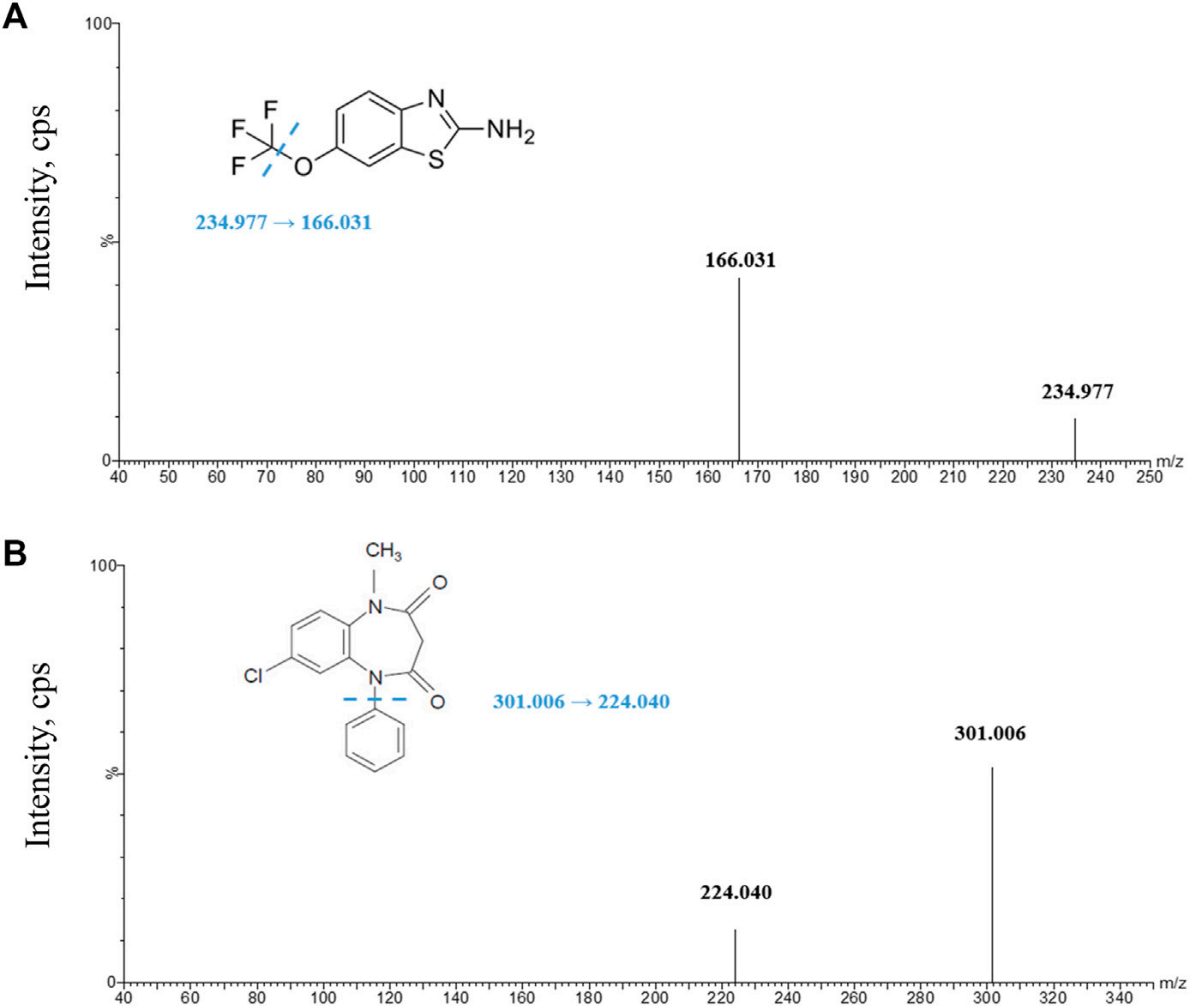
Product ion spectra of analytes. **(A)** Riluzole parent ion and daughter ion scan; **(B)** clobazam parent ion and daughter ion scan. The illustrations in Figures **(A)** and **(B)** give the structures of riluzole and clobazam, respectively, and the location of the possible break.

Riluzole (Figure 1) was purchased from MedChemExpress (New Jersey, United States). The internal standard (IS) clobazam (Figure 1) (solution in methanol, 100 μg/mL, expiry date 2024.2.23) was purchased from Tianjin Alta Technology Co., Ltd. (Tianjin, China). Methanol, acetonitrile, and water (HPLC-grade) were purchased from Honeywell Burdick and Jackson (Ulsan, Korea). Formic acid (analytical grade) was purchased from Thermo Fisher Scientific CN Co., Ltd. (Shanghai, China).

### 2.2 UPLC conditions

Chromatographic separation was performed on an ACQUITY UPLC I-Class System (Waters, Milford, United States). The analytical column was an ACQUITY UPLC^®^ HSS T3 (2.1 × 50 mm, 1.8 μm, Waters, Milford, United States). The column temperature was set to 40°C. The mobile phase consisted of 0.1% formic acid in acetonitrile and 0.1% formic acid in water. The gradient elution procedure started with 95% formic acid in water maintained for 0.5 min, then the gradient was decreased to 40% formic acid in water within 0.5 min. In addition, the proportion of formic acid in water was continuously decreased to 5% within 2 min and held for 1 min. Finally, the proportion of formic acid in water was switched back to 95% in 0.5 min and held for 0.5 min before the next run. The flow rate was fixed at 0.3 mL/min. In addition, riluzole and clobazam were gradient-eluted at 1.9 min and 2.12 min, respectively. The injection volume was 5 μL, and the total running time was set to 5 min.

### 2.3 Mass spectrometer conditions

Mass spectrometry was performed using a Xevo TQ-S mass spectrometer (Waters, Milford, United States) equipped with a positive-mode electrospray ionization source (ESI). The test was operated in a multiple-reaction monitoring (MRM) mode with a dwell time of 0.043 s per conversion. Due to time and price constraints, we chose clobazam, which was already available in the laboratory and has similar UPLC conditions to those of riluzole, as the internal standard. The transitions monitored were m/z 234.977→166.031 for riluzole and m/z 301.006→224.040 for clobazam. Ultrahigh-pressure nitrogen was used as the desolvation gas (1000 L/h) and the cone gas (150 L/h). The desolvation temperature was 500°C. The collision energy of riluzole and IS was 28 V and 32 V, respectively. In addition, the capillary voltages of riluzole and IS were both 3 kv.

### 2.4 Calibration standards and quality control samples

In order to save the drug-free human plasma, we first dilute the stock solution into the working standard solutions and QC solutions with reagents and then dilute the working solutions and QC solutions into the standards and QC samples with the drug-free plasma. The working standard solutions were prepared at concentrations of 0.5, 1, 2, 4, 10, 20, 40, and 80 μg/mL by diluting the stock solution (1 mg/mL) with water–acetonitrile (v: v = 95:5). A standard curve of final concentrations of 5, 10, 20, 40, 100, 200, 400, and 800 ng/mL was obtained by diluting the working standard solution with drug-free human plasma. Similarly, QC solutions at concentrations of 1.5, 6, and 60 μg/mL were also obtained by diluting the stock solution with water–acetonitrile (v: v = 95:5). Quality control samples (QCs) at concentrations of 15, 60, and 600 ng/mL were also obtained by dilution of blank human plasma. All solutions, standards, and QCs were stored frozen at −80°C.

### 2.5 Sample preparation

A 50 μL volume of the plasma sample was first added to a 1.5 mL Eppendorf tube, and then 200 μL protein precipitation solution (acetonitrile) containing IS (5 ng/mL) was added. The mixture was thoroughly vortexed for 2 min. The supernatant (50 μL) was collected by centrifugation at 12,000 rpm for 10 min. Then, 450 μL of water–acetonitrile (v: v = 95:5) was added and mixed well. The plasma volume used (50 μL) is much lower than that of the published studies (200 μL–1 mL) (Sarkar et al., 2017; Chandu et al., 2010; Chow et al., 2012; Le Liboux et al., 1997; van Kan et al., 2004).

### 2.6 Data acquisition and analysis

All data were collected and processed using MassLynx software (version 4.1 SCN810). A weighted least-squares linear regression model (weighted 1/x2) was used to establish the relationship between the concentration and the peak area ratio of the analyte to IS. The concentrations of the QC samples and the unknown clinical sample were calculated by interpolation of the equation. For QC samples and calibration standards, except for LLOQ samples (bias <20%), the remaining sample bias should be <15%. In addition, in each analysis run, more than 75% of the QC samples should meet the aforementioned criteria, and the correlation coefficient (r) should be >0.99.

### 2.7 Method validation

The method was validated in accordance with the FDA (2018) bioanalytical method validation guidance for industry and the European Medicines Agency (2011) guideline on bioanalytical method validation including precision and accuracy, LLOQ, linearity, and stability of the analyte under various conditions, such as carry-over, extraction recovery, and matrix effect.

Three independent and continuous batches of LLOQ, low-, medium-, and high-QC samples were evaluated in at least 2 days to assess the precision and accuracy. In each analysis batch, the QC assessment for each concentration level was repeated six times. Relative standard deviation (RSD) and relative error (RE) were used to indicate precision and accuracy, respectively. In addition to LLOQ, the other three concentration levels of QCs’ intra-batch and inter-batch RSD should be *≤ 15%* and RE should be within *±15%*, while LLOQs’ inter-batch and intra-batch RSD should be <*20%* and RE should be within *±20%*.

The linearity response of riluzole was assessed over calibration ranges. The calibration curve was performed with eight concentrations, and the requirements were the same as those given in Section 2.6.

Stability assessment of the riluzole in a stock solution included long-term stability and short-term stability. However, according to the certificate of analysis (COA) of riluzole, the riluzole solution can be stored for up to 6 months at −80°C, and the short-term stability was evaluated by keeping it at room temperature for 5 h. The IS was diluted when it would be used.

Stability assessment of the analyte in plasma and whole blood included short-term stability, autosampler stability, freeze–thaw stability, whole blood stability, and long-term stability. Short-term stability was evaluated by measuring QCs maintained at room temperature for 4 h. Autosampler stability was achieved by analyzing the processed QCs that had been held in the autosampler (10°C) for 24 h. The freeze–thaw stability was evaluated after seven freeze (−80°C) and thawing (room temperature) cycles before QC sample preparation. Whole blood QCs were kept at room temperature for 4 h to assess the whole blood stability. Long-term stability was assessed by analyzing QCs after storage for 26 days at −80°C.

Extraction recovery was evaluated by comparing the peak areas of the analyte extracted from the three-level QC samples with those of post-extraction spiked QC samples at the same concentrations. The RSD of the extraction should be *≤ 15%*; the RE should be within *±15%*.

Matrix effects were assessed using low-, medium-, and high-level QCs from six individuals. Matrix factors were evaluated by calculating the peak area’s ratio of the presence of the matrix (measured by analyzing the blank matrix spiked with the analyte after extraction) to the absence of the matrix (pure solution of the analyte). The RSD of the matrix effect should be *≤ 15%*.

### 2.8 Application

The validated analytical method was applied to determine the concentration of riluzole in patient plasma after oral administration of riluzole. The clinical study was approved by the Ethics Committee of Peking Union Medical College Hospital and was in line with good clinical practice. All subjects signed informed consent prior to the study. The criteria for inclusion of ALS patients taking riluzole in our hospital were as follows: 1) age ≥18 years; 2) the treatment course of oral riluzole was ≥2 weeks, indicating that riluzole reached homeostasis in the patient. Exclusion criteria: 1) the duration of medication could not be determined; 2) elderly patients (≥80 years of age) with poor basic condition and many influencing factors are difficult to evaluate; 3) had taken clobazam within 1 month. Since riluzole reached a steady state in enrolled patients, a blood sample was collected from each patient at a random time point, and the last time of medication before blood collection, the time of blood collection, and demographic information about the patients were accurately recorded. All blood samples were centrifuged at 3000 rpm for 10 min to obtain plasma samples, which were stored at −80°C prior to analysis.

## 3 Results and discussion

### 3.1 Method development and optimization

#### 3.1.1 Optimization of mass spectrometric parameters

Filtering and optimizing mass spectrometry conditions by systematic methods. We tried atmospheric pressure chemical ionization (APCI) and ESI source in positive and negative ionization modes. Usually, positive ionization is the preferred ionization mode for the detection of neutral or basic analytes. The results show that the ESI source has higher ionization efficiency in the positive ionization mode, which may be related to the presence of the amine group in the molecular structure of riluzole.

An apposite MRM transition of riluzole and IS was selected based on the ion spectrum of the analyte product. In addition, the voltage and gas parameters were adjusted to obtain the most suitable ionization and fragmentation conditions.

#### 3.1.2 Optimization of chromatographic conditions

In order to improve the response and peak shape and reduce the carryover of riluzole, the organic phase and aqueous phase of formic acid with different proportions were tested. Finally, it was found that when the mobile phase was 0.1% formic acid in acetonitrile and 0.1% formic acid in water, and the flow rate of the system was 0.3 mL/min, the best effect was obtained. In addition, under the chromatographic conditions described herein, effective chromatographic separation guaranteed the accurate determination of riluzole within a running time of 5 min.

#### 3.1.3 Optimization of sample preparation

Protein precipitation, liquid–liquid extraction, and SPE are the most common sample pre-treatment methods. Previously reported pre-treatment methods of riluzole samples were liquid–liquid extraction or SPE, which were considered too complicated. Therefore, our study explored the selection of plasma volume, the proportion of the precipitant to plasma, and whether to dilute the supernatant. The results showed that the response of riluzole was appropriate when the volume of the plasma sample was 50 μL, the acetonitrile-to-plasma ratio was 4:1, and 50 μL of the supernatant was diluted with 450 μL solution. If the supernatant was not diluted, the response of the ULOQ sample (800 ng/mL) will exceed the highest response of the machine. In the process of exploration, in order to save clinical samples, we tried to take only 5 μL plasma and added 20 μL acetonitrile in the ratio of 1:4 for protein precipitation, but the standards and QCs’ bias did not meet the requirements. In addition, the dilution of the supernatant with different proportions of water–acetonitrile and whether to add 0.1% formic acid were studied. The response of riluzole was appropriate when water–acetonitrile (v: v = 95:5) was chosen as a diluent, and there was no effect on the results irrespective of adding 0.1% formic acid in water–acetonitrile (v: v = 95:5), so we chose the simpler water–acetonitrile (v: v = 95:5) as a diluent.

In addition, we tried different collision energies, and each analyte obtained three different daughter ions, namely, m/z 234.977→166.031 (28 V), 234.977→149.764 (30 V), and 234.977→138.053 (34 V) for riluzole and m/z 301.006→224.04 (32 V), 301.006→153.064 (36 V), and 301.006→105.110 (34 V) for clobazam. We compared their response, peak shape, and carryover after sample preparation under the corresponding chromatographic conditions. Finally, the product ion spectra were obtained (Figure 1) to select the transitions of the analyte (riluzole, 234.977→166.031) and the IS (clobazam, m/z 301.006→224.040).

All in all, the time and effort required to prepare samples using protein precipitation is significantly reduced compared to that taken by liquid–liquid extraction or SPE reported so far. In addition, the relatively short running time of the method (5 min) and the plasma volume used (50 μL) is much smaller than that of the published works (200 μL–1 mL), making it ideal for drug analysis in the clinical setting (Sarkar et al., 2017; Chandu et al., 2010; Chow et al., 2012; Le Liboux et al., 1997; van Kan et al., 2004).

### 3.2 Method validation

#### 3.2.1 Linearity, carryover, and selectivity

Eight calibration standards were analyzed per batch, and the calibration curve showed good linearity in the range of 5–800 ng/mL. The regression coefficients of all standard curves were >0.99 and the back-calculated concentrations were within *85%–115%* of their nominal values (Table 1). Carryover was investigated by testing the blank sample after the upper limit of quantitation (ULOQ). The peak area of carryover was within *20%* of the peak area at LLOQ in every analytical batch. As a result, the peak area of carryover was approximately *10%* of the peak area at LLOQ throughout method validation, which met the requirements.

**TABLE 1:** Back-calculated concentrations of calibration standards for riluzole (linear-weighted 1/x2).

Item	S1 (n = 15)	S2 (n = 15)	S3 (n = 15)	S4 (n = 13)	S5 (n = 16)	S6 (n = 13)	S7 (n = 16)	S8 (n = 16)	*R* ^2^	Slope	Intercept	Regression equation
Nominal	5.00 ng/mL	10.0 ng/mL	20.0 ng/mL	40.0 ng/mL	100 ng/mL	200 ng/mL	400 ng/mL	800 ng/mL				
Concentration												
Mean^a^	4.95	9.55	19.3	42.8	94.7	215.9	426.2	765.3	0.996	0.972	5.994	y = 0.972x + 5.994
RE (%)^b^	−0.9	−4.5	−3.7	7.0	−5.3	7.9	6.5	−4.3				
RSD (%)^c^	3.8	3.4	4.7	5.6	3.2	4.2	3.2	2.5				

^a^ Concentration is to three significant figures.

^b^ Expressed as [(mean observed concentration − nominal concentration)/(nominal concentration)] × 100 (to one decimal place).

^c^ Relative standard deviation: standard deviation/mean × 100 (to one decimal place).

**FIGURE 2:**
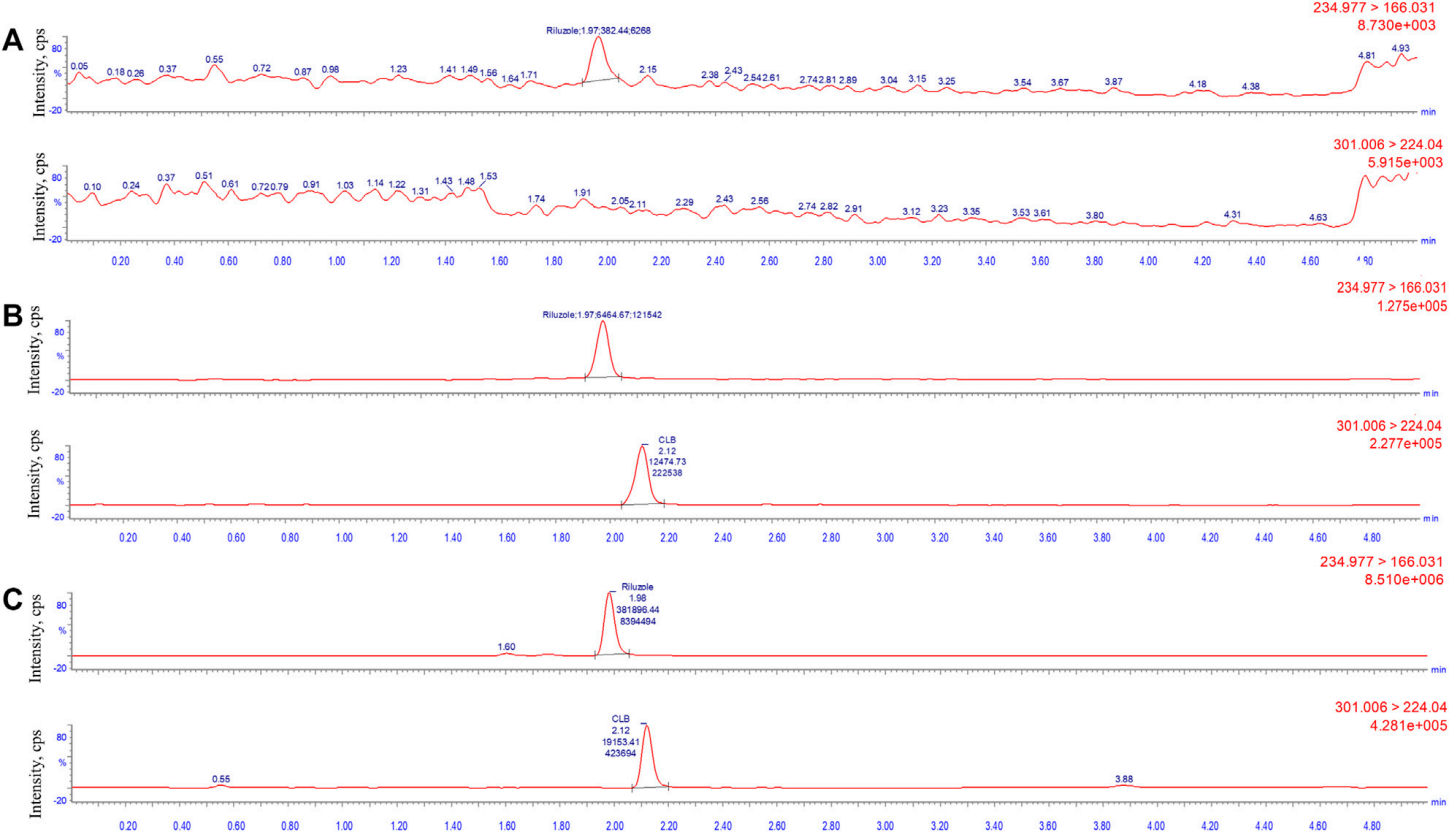
Typical multiple-reaction monitoring chromatograms of riluzole and clobazam: **(A)** blank plasma sample; **(B)** plasma spiked with the analytes at LLOQ level (5 ng/mL) and internal standard; and **(C)** real clinical samples (137.2 ng/mL).

The concentration of LLOQ was 5 ng/mL. Figure 2 shows a typical chromatogram of the analyte and IS in blank plasma samples spiked with the analyte at the LLOQ level and IS (5 ng/mL). The results indicated that the method had satisfactory selectivity for riluzole and IS.

#### 3.2.2 LLOQ, precision, and accuracy

Table 2 summarizes the intra- and inter-assay precision and accuracy of riluzole measured by LLOQ and three QC levels. Compared with the nominal concentration, the RE of LLOQ samples ranged from *-12.3% to 5.9%*, the RE of other QC samples ranged from *-10.1% to 13.1%*, and the RSD level for each concentration was also *<15%*. The results showed that the precision and accuracy were within an acceptable range. The method was reliable and reproducible and can be used for the determination of riluzole concentration in human plasma.

**TABLE 2:** Intra- and inter-day precision and accuracy of quality control samples of riluzole.

Batch	Item	LLOQ	Low QC	Medium QC	High QC
	Nominal concentration	5.00 ng/mL	15.0 ng/mL	60.0 ng/mL	600 ng/mL
1 (n = 6)	Mean^a^	5.1	15.5	55.6	632
1 (n = 6)	RE (%)^b^	2.0	3.4	−7.4	5.3
1 (n = 6)	RSD (%)^c^	2.5	1.9	1.4	2.9
2 (n = 6)^d^	Mean^a^	5.1	15.8	54.6	629
2 (n = 6)^d^	RE (%)^b^	2.3	5.0	−8.9	4.9
2 (n = 6)^d^	RSD (%)^c^	2.9	2.7	0.8	0.7
3 (n = 6)	Mean^a^	4.5	15.4	63.1	660
3 (n = 6)	RE (%)^b^	−11.0	2.6	5.2	10.0
3 (n = 6)	RSD (%)^c^	1.9	1.7	2.0	1.6
Inter-day (n = 17)	Mean^a^	4.9	15.6	58.0	640
Inter-day (n = 17)	RE (%)^b^	−2.2	3.7	−3.4	6.7
Inter-day (n = 17)	RSD (%)^c^	6.9	2.3	6.9	2.9

^a^ Concentration is to three significant figures.

^b^ Expressed as [(mean observed concentration − nominal concentration)/(nominal concentration)] × 100 (to one decimal place).

^c^ Relative standard deviation: standard deviation/mean × 100 (to one decimal place).

^d^ For batch 2, the number of Q2 was 5.

#### 3.2.3 Matrix effect and extraction recovery

The results of matrix effect and extraction recovery are shown in Table 3. The matrix effect of riluzole was between *99.1% and 107.6%*; therefore, the matrix did not significantly affect the determination of drug concentration. The overall average extraction recovery of riluzole at three different concentrations was *97.2%*.

**TABLE 3:** Matrix effect (n = 6) and extraction recovery (n = 6) of riluzole in plasma.

	Spiked concentration (ng/mL)	Extraction recovery (%)		Matrix effect (%)	
		Mean ± SD	RSD	Mean ± SD	RSD
Riluzole	15.0	93.0 ± 3.9	4.2	103.8 ± 3.6	3.5
	60.0	100.1 ± 1.8	1.8	102.4 ± 0.7	0.7
	600	98.5 ± 1.4	1.4	105.2 ± 0.6	0.6
Clobazam	5.00	109.6 ± 4.7	4.3	103.7 ± 2.9	2.8

^a^ Concentration is to three significant figures.

^b^ Expressed as [(mean observed concentration − nominal concentration)/(nominal concentration)] × 100 (to one decimal place).

^c^ Relative standard deviation: standard deviation/mean × 100 (to one decimal place).

#### 3.2.4 Stability

The results showed that the riluzole stock solution can be kept at room temperature for 4 h. The stability results of riluzole in plasma and in whole blood are shown in Table 4. The riluzole plasma samples could be stably placed at room temperature for 4 h, remained stable after seven freeze–thaw cycles (−80°C to room temperature), and could be stably stored at −80°C for at least 26 days. In addition, the processed samples remained stable after being placed in the autosampler for 24 h, and the riluzole whole blood samples were stable for 4 h at room temperature, providing a time window for clinical sample processing. At the same time, according to the concentrations measured after the preparation of the whole blood QCs, it can be inferred that riluzole may be distributed in the whole blood cells, but further experimental verification is needed.

**TABLE 4:** Stability assessments for riluzole in plasma, processed, and whole blood samples (n = 6).

Stability type	Nominal concentration (ng/mL)	Mean^a^	RE (%)^b^	RSD (%)^c^
Short-term	15.0	14.8	−1.3	1.5
(plasma samples, room temperature for 4 h)	60.0	62.3	3.8	1.6
	600	630.8	5.1	1.4
Freeze–thaw	15.0	15.3	1.9	1.3
(plasma samples, seven cycles)	60.0	61.8	3.0	1.0
	600	635.3	5.9	1.6
Long-term	15.0	16.8	11.9	1.3
(plasma samples, −80°C for 26 days)	60.0	66.4	10.6	0.9
	600	677.9	13.0	1.6
Autosampler	15.0	17.2	14.8	1.9
(processed samples, 10°C for 24 h)	60.0	66.9	11.6	1.2
	600	685.4	14.2	1.7
Whole blood^a^	7.1	7.0	−0.9	1.6
(room temperature for 4 h)	29.3	25.6	−12.6	1.2
	279	275	−1.5	1.3

^a^ Concentration is to three significant figures.

^b^ Expressed as [(mean observed concentration − nominal concentration)/(nominal concentration)] × 100 (to one decimal place).

^c^ Relative standard deviation: standard deviation/mean × 100 (to one decimal place).

*When configuring whole blood stable QC samples, assuming that riluzole is not distributed in blood cells, the volume of plasma centrifuged is about half of the volume of whole blood, so the configured final concentrations of whole blood QC samples were 7.5, 30, and 300 ng/mL.

#### 3.2.5 Application

The UPLC-MS/MS method was successfully applied to determine the steady-state concentration of riluzole in ALS patients treated with riluzole for at least 1 month. So far, 10 plasma samples from 10 ALS patients have been successfully analyzed, and no notable problems have occurred during the whole analysis, such as changes in retention time and interference. Table 5 shows the concentrations of 10 subjects who received riluzole. At present, steady-state trough concentrations were collected for patients 2, 4, 8, 9, and 10, and the collection time points for other patients were in the elimination phase. According to (Bruno et al. 1997), the steady-state trough concentration of patients with SCI after taking riluzole ranged from 11.17 to 147.55 ng/mL. The concentrations of the five samples that had reached the steady state in our experiments were 20.6, 37, 16, 37.4, and 58 ng/mL, respectively, which were consistent with the range of SCI patients. Of course, further verification of large samples was needed. Therefore, based on the current results, 5 ng/mL was a suitable LLOQ. At the same time, considering the range of steady-state peak concentration of riluzole reported in foreign patients with SCI (Bruno et al., 1997), the ULOQ is also appropriate in order to continue the determination of subsequent collected samples, including those with around peak concentration. In general, the range of the standard curve in our method was suitable for the clinical setting. We will further establish popPK of riluzole in Chinese ALS patients after measuring the samples collected later and compare its pharmacokinetic characteristics with those of foreign patients with ALS or SCI (Bruno et al., 1997).

**TABLE 5:** Concentration of riluzole in ALS patients after multiple oral doses (50 mg bid).

Patient	The time of the last dosing before the blood collection (h)	Concentration (ng/mL)	Patient	C _trough_ (ng/mL)
1	4.5	37	2	20.6
3	4	76.6	4	37
5	8.5	71.7	10	16
6	2.5	137.2	11	37.4
7	5.25	90.8	13	58

Note: Steady-state trough concentrations were collected for patients 2, 4, 8, 9, and 10.

In addition, as the disease progresses, swallowing difficulties may gradually appear in ALS patients, resulting in an inability to take oral riluzole tablets. Therefore, in recent years, researchers have devoted themselves to the development of new dosage forms of riluzole, such as suspension and oral films (Wymer et al., 2023). The simple method was established for the determination of riluzole concentration, which was convenient for other groups to refer to, and can be applied in the study of pharmacokinetics and bioavailability of new forms of riluzole.

## 4 Conclusion

A robust UPLC-MS/MS method has been developed and validated for the determination of riluzole in human plasma, and the feasibility of this method was further verified by the determination of the riluzole concentration in clinical samples. Compared with other reported analytical methods, our method has the advantages of simple sample preparation, relatively short running time, and small plasma sample volume. Considering the high-throughput potential of our method, it can be easy to analyze patient samples in the clinical setting or clinical trials. At the same time, this was the first published method used to determine the riluzole concentration of Chinese ALS patients, and the first to report the stability data of riluzole in whole blood, which is conducive to the development of subsequent studies related to the pharmacokinetics of riluzole.

## Data Availability

The raw data supporting the conclusion of this article will be made available by the authors, without undue reservation.
